# The knowledge, attitudes and beliefs of midwives on the vaccination coverage rates in Perth’s Aboriginal children

**DOI:** 10.1186/s12889-021-11907-1

**Published:** 2021-10-12

**Authors:** Rebecca Carman, Lesley Andrew, Amanda Devine

**Affiliations:** 1 School of Nursing and Midwifery, Edith Cowan University, Perth, Australia; 2 School of Medical and Health Sciences, Edith Cowan University, Perth, Australia

**Keywords:** Aboriginal, Indigenous, Children, Immunisation, Vaccination, Midwife, Midwives, Mixed methods, Primary research, Immunization

## Abstract

**Background:**

Midwives are well placed to promote vaccination awareness throughout a women’s pregnancy and strengthen childhood vaccination demand following hospital discharge. In Perth, Western Australia, Aboriginal children experience some of the lowest vaccination coverage rates across the nation. To identify factors preventing greater vaccination uptake amongst the target population, a theory-based study was conducted with midwives across two Perth maternity hospitals to explore behavioural attributes, knowledge, attitudes and beliefs surrounding vaccination provision and the vaccines administered to Aboriginal children.

**Methods:**

A purpose-designed questionnaire was distributed to midwives working in two Perth public maternity hospitals. The proximal constructs of The Theory of Planned Behavior were used to frame the questionnaire to enable the barriers to greater vaccination coverage to be identified and behaviourally situated. Descriptive statistics described the demographics of the study sample. Chi-square and the Fisher’s exact test were used to identify associations between midwife characteristics and awareness of the coverage rates. Significance was set at α = 0.05.

**Results:**

Of the 58 midwives who completed the study questionnaire, 77.2% were unaware of the sub-optimal vaccination coverage in Perth’s Aboriginal children. Level of education (*p* = 0.53) and years worked as a practising midwife (*p* = 0.47) were not found to be associated with an awareness of the coverage rates. Approximately, 50% of midwives reported some concern over the efficacy of childhood vaccines, 44.4% did not feel confident with their knowledge of vaccines, while 33.3% do not routinely discuss childhood vaccinations with parents prior to hospital discharge.

**Conclusions:**

Key findings in the study identified that a range of educational, leadership and system-based issues are affecting midwives’ capacity to play a more substantial role in influencing vaccination coverage in Perth’s Aboriginal children.

## Background

Discussions on vaccine application and use are often initiated by healthcare providers during a women’s pregnancy [1, 2]. This practice has been shown to enhance vaccine uptake and reduce parental vaccine hesitancy particularly if it has been recommended by a healthcare provider [1]. For midwives who practice a women-centred approach, which is designed to empower and enable women to make informed decisions, providing the most current evidence available, is fundamental to their role. However, while it is globally well established that midwives are a critical and trusted source of patient-education [1–3], tension in midwives may arise when advocating vaccines if there are concerns surrounding vaccine safety [1, 3] or deficits in their vaccine knowledge when providing patient education [1]. Internal conflict may also occur in midwives who have vaccination beliefs that contrast with information they are required to provide to new parents by their regulatory body (Nursing and Midwifery Board of Australia [NMBA]) particularly if it is perceived to reduce a mother’s available options [1].

A 2018 study conducted in France [2] explored midwife attitudes to vaccination practice. More than one-half of all midwives in the study felt they were inadequately trained in and informed about vaccines. Similarly, an Australian study [4] suggested that vaccination content delivered in midwifery undergraduate degrees by local universities was most likely inadequate. Despite midwives being one of the most commonly accessed healthcare providers that pregnant women discuss vaccinations with, a considerable proportion of parent’s report that inadequate vaccine information is provided during the perinatal period [3, 5, 6]. This can result in certain downstream effects such as parental uncertainty as they contemplate vaccination during their child’s early years.

Workplace education to inform vaccination provision in the clinical setting is an important strategy to increase pre-existing vaccine knowledge [1, 3]. Expanding knowledge in this area for midwives has been shown to positively impact the outcomes of difficult vaccination discussions with parents, particularly in those who display vaccine hesitancy [6]. Previous studies have identified that it is during the ante-natal period that parents start to ask questions regarding vaccination and similarly are requesting a broad range of information be provided on childhood vaccines [6]. Commonly cited barriers most often reported by pregnant women and parents which impact decisions to vaccinate their children include inadequate knowledge on the benefits of vaccines, uncertainty of the perceived risk of getting a disease, and safety issues [6]. Given that midwives have frequent contact with parents during the ante-natal period which builds trust and rapport, information that is considered acceptable and easily understood is likely to increase vaccine uptake (Hassen et al., 2019).

Specific barriers associated with vaccine advocacy and promotion by midwives are described in the literature [1–3]. Some midwives report not having additional opportunities to bolster their vaccine knowledge in the workplace after completing their undergraduate degrees [1, 3]. Midwives also describe having to undertake their own research or rely on information received when they get their own children vaccinated [1]. The perception that vaccine administration is outside their scope of practice, not wanting to add further pressure to pregnant women or referring women on to mainstream resources (for example, online websites) to gain vaccine knowledge is also evident in the literature [5]. From a global perspective, the specific vaccines administered by midwives in the hospital setting varies [1, 3].

In Western Australian (WA) birthing hospitals, ward-based midwives are authorised to administer the hepatitis B vaccine to infants within 7 days of their birth. This is the first vaccine administered in the Western Australian (WA) childhood immunisation schedule [7]; it also signals the start of a child’s vaccination journey. All other childhood vaccines subsequent to this are delivered from 6 weeks of age to 4 years and typically occur after hospital discharge following a birth. For Aboriginal children living in WA who are at greater risk of acquiring certain vaccine preventable diseases, additional vaccines are prescribed during these childhood years. However, despite national improvements seen in childhood vaccination uptake across Australia, Aboriginal children located in Perth, WA are reported to have some of the lowest rates of vaccination coverage across the nation [7]. This is bench-marked against the national target (95%) and of *all children* in the same location. At study commencement, the coverage rates of Perth-based Aboriginal children aged 12–15 months (2018) were 86.76%; at 24–27 months, this figure was 78.75% [7]. Encouragingly, by 60–63 months, a high proportion of Aboriginal children (94.44%) were fully vaccinated. In contrast, 93.86% of *all children* in Perth at 12–15 months were fully vaccinated, 89.57% at 24–27 months and 92.93% at 60–63 months.

Improvements in vaccination coverage in Aboriginal children at 60–63 months are reassuring. They are also an acknowledgement of the significant efforts made by parents, healthcare providers, and changes to the eligibility of government payments provided to parents for vaccinating their children. However, health disparities between Aboriginal and non-Aboriginal children in Perth persist [8–10]. Addressing the disparity of health in Aboriginal children as a result of vaccine-preventable disease presents an opportunity for healthcare providers to modify this gap. The literature reports that vaccine-preventable diseases seen in Aboriginal children are over four times that of non-Aboriginal children [9]. To provide local context, this finding is further compounded by a reported over-representation of Aboriginal children presenting to Perth metropolitan Emergency Departments (ED) [8]; many of these ED presentations are attributed to vaccine-preventable respiratory infections.

Previous studies describing the attitudes of midwives towards childhood vaccination indicate that the majority are supportive of the benefits and provision [1, 2, 8]. Midwives also believe that vaccines should be discussed with parents during an inpatient stay; however whether this consistently occurs or in what depth is somewhat unclear [11]. An Australian study [2] concluded that midwives were more likely to provide effective education to parents if they had completed additional vaccination training. Similarly, Vishram et al. [6] suggested that clinical staff with dedicated vaccination education were more confident in providing vaccine advice to pregnant women and managing appropriate responses to parental concerns. The literature also suggests that childhood vaccination uptake is higher in women who have received vaccine recommendations by a midwife or other healthcare provider’s during their pregnancy [2]. The aim of this study was to explore the knowledge, attitudes, and behavioural attributes of hospital-based midwives in Perth surrounding vaccination provision in Aboriginal children to identify barriers that could be preventing greater vaccination coverage in the studied population.

## Methods

### Questionnaire

A pre-piloted questionnaire [12] based on theoretical framework [13] was used to identify the behavioural barriers in the vaccine-associated practise of midwives working in two maternity hospitals in the Perth metropolitan area. The proximal constructs of the Theory of Planned Behavior [13] were used to provide framework in the development of the 54 item questionnaire. The questionnaire was distributed via email by a hospital-based Clinical Nurse Manager (CNM) in each hospital site (*n* = 2) using a live online survey link in Qualtrics to 100 midwives. The Participant Information and Consent Form (PICF) were embedded into the online questionnaire and midwives had to indicate that they had read the study information and consented to participate prior to commencing the questionnaire. Due to a low online questionnaire response rate (*n* = 16, 16%) which occurred despite reminder emails being sent to participants at two and at 3 weeks following initial circulation, hard copy questionnaires were distributed. All ethical approvals including questionnaire amendments for hard copy distribution were sought and received prior to study commencement. Methods were performed in accordance with the relevant guidelines and regulations associated with the approvals provided.

### Theoretical framework for questionnaire

The four proximal constructs of the *Theory of Planned Behavior* (TPB) were used to frame the questionnaire [13]. The theory posits that the four proximal constructs (attitude, perceived behavioural control, subjective-norm, and intention) are strong predictors of an outcome behaviour occurring. In the current study, the outcome behaviour is awareness of the coverage rates in the studied population. Statements relating to vaccine provision in Aboriginal children were input into each of the proximal constructs.

A person’s ‘attitude’ is said to refer to the degree to which a positive assumption of a behaviour is held [13]. For example, whether a midwife believes that vaccines are safe may influence whether she promotes a vaccine. A person who perceives a behaviour to be favourable is also more likely to consider performing the behaviour. The ‘subjective-norm’ construct relates to whether the person feels that a specific behaviour is looked upon favourably by peers, colleagues, and persons of considered importance. If a midwife perceives the (hospital) ward based CNM to view vaccines as a priority this may influence the midwife to champion vaccines and be more proactive in the promotion of vaccines during a women’s hospital stay. The construct, ‘perceived behavioural control’ refers to an individual’s perception of how easy or difficult an action or behaviour is to perform or complete; while intention is influenced by all other constructs and collectively refers to the motivational factors that influence whether a behaviour is performed.

### Questionnaire instrument

The questionnaire was developed following a comprehensive search of the literature, formal discussions with informed Aboriginal and non-Aboriginal health professionals, and using the proximal constructs of the TPB to provide structure [13]. To ensure that the questionnaire content was culturally relevant and appropriate, consultation took place with members of the Aboriginal community working in local and interstate government health departments, a national Aboriginal vaccine researcher and personnel from the National Aboriginal Community Controlled Health Organisation (NACCHO). Piloting of the questionnaire was also undertaken (*n* = 10) prior to distribution to provide content and face validity [12]. This was undertaken with informed health professionals: medical, science and nursing university academics, a general practitioner, an epidemiologist in the WA Department of Health, an Aboriginal Child Health Manager, Aboriginal Health Officer, and clinical management staff working in Child and Adolescent Health in the WA Department of Health.

To gauge level of agreement to statements within each of the four proximal theory constructs, a five-point Likert scale was used. The Likert responses ranged from *Strongly agree* to *Strongly disagree*. Data was analysed using the five-point scale, but in table presentation, collapsed to *Agree, Uncertain* and *Disagree*. In addition to the statements used within each of the theory constructs, the questionnaire explored midwives’ perceptions of barriers to a more efficient vaccination service using binary response (Yes or No).

### Questionnaire participants

Participants were midwives working in two Perth-based maternity hospitals which record high numbers of Aboriginal women giving birth in each year. A pre-determined sample size was not calculated prior to study commencement. It is well established that obtaining an adequate sample size in health professionals is methodologically challenging [14]. Factors such as a general lack of time, concerns with the confidentiality of their responses, and the perceived value of their input have all been documented as reasons contributing to low response rates.

A personalised email containing the questionnaire link was circulated by a CNM working in each hospital to their practising midwives. After 2 weeks following initial questionnaire dissemination, a reminder email was sent to staff to encourage commencement or completion; a second email reminder was sent at week three. Due to an initial low questionnaire response rate (16%), an amendment was submitted to the Human Research Ethics Committee’s (HREC) requesting approval for hard copy questionnaire distribution. Hard copy versions of the survey were delivered in-person to each hospital by the first author (RC) and given to the hospital contacts (CNM) for distribution. Completed hard copy surveys were returned by midwives to the hospital CNM in each site and stored in a locked drawer until collection by RC (in-person) occurred. This strategy resulted in a combined hospital total of 58 completed questionnaires (54 items).

### Questionnaire statistical analysis

The completed online and hard copy questionnaire data were combined in the online platform Qualtrics before being imported into IBM SPSS Version 26 (IBM, New York, USA). Questionnaire responses were coded, and then statistically analysed. Descriptive statistics described the characteristics of the study sample and are displayed throughout as a count, and as percentages.

To determine the association between awareness of the vaccination rates in Aboriginal children and midwife characteristics, the chi-square test and Fishers exact tests were used. For example, whether total years working as a midwife, or level of education were associated with awareness of the low vaccination coverage observed in Perth’s Aboriginal children. Imported data was analysed in SPSS using the five-point Likert scale which ranged from Strongly agree to Strongly disagree. For certain statements where the observed count received was < 5, variables were collapsed; where this occurs, a notation in the results has been made.

### Ethics approval and consent to participate

Ethical approval to conduct this study was received from the Edith Cowan University (ECU) Human Research Ethics Committee (HREC) (#18089), Sir Charles Gairdner and Osborne Park Healthcare Group HREC (#00602), the Western Australian Aboriginal Health Ethics Committee (WAAHEC) (#827), and the St John of God (SJOG) Healthcare HREC (#1289). A letter of support was also sought and received from the Derbarl Yerrigan Health Service (DYHS); this was a requirement of the WAAHEC approval.

Governance approval was obtained from the WA Department of Health and SJOG Healthcare. This enabled access to staff across the two hospital sites. Informed consent was received from all online questionnaire participants using the Participant Information and Consent Form (PICF) which was embedded into the online questionnaire. Written informed consent was received by all participants who completed the hard copy questionnaires; this occurred prior to commencement of data collection.

## Results

### Questionnaire: demographics

The majority of midwives working across the two Perth-based birthing hospitals cited a graduate diploma or graduate certificate (*n* = 31, 54.4%) as their highest level of education achieved; 17.5% (*n* = 10) had a bachelor undergraduate degree, while 12.3% (*n* = 7) reported having an undergraduate diploma. None of the midwives who responded the questionnaire had undertaken a PhD. Almost two-thirds of the midwives (*n* = 35, 62.5%) had been administering vaccinations to children for more than 10 years and all but one was female (see Table 1).

**Table 1:** Demographics of the study sample

Question	n*	Response Options	Frequency	%
What is the highest level of education achieved?	57	Hospital based training	5	8.8
		Diploma	7	12.3
		Bachelor’s degree	10	17.5
		Graduate certificate/ diploma	31	54.4
		Master’s degree	3	5.3
		Doctoral degree (PhD)	0	0
		Other	1	1.8
How long have you been providing vaccinations to children?	56	< 6 months	2	3.6
		6 - < 12 months	1	1.8
		1 - < 5 years	8	14.3
		5 - ≤10 years	10	17.9
		> 10 years	35	62.5
How frequently do you vaccinate Aboriginal children?	57	Every day	1	1.8
		Once per week	16	28.1
		Once per month	13	22.8
		Once every few months	8	14.0
		Rarely	13	22.8
		Not at all	6	10.5
What is your gender?	57	Female	56	98.2
		Male	1	1.8

### Questionnaire: cultural safety and vaccination awareness

More than three-quarters of all midwives who responded to the questionnaire (*n* = 44, 78.6%) had completed annual cultural safety training within their setting. When midwives were asked whether they were aware of the low coverage rates in Perth’s Aboriginal children, only 22.8% (*n* = 13) reported having knowledge in this area. Level of education achieved by midwives was not found to be associated with awareness of the low vaccination coverage rates (*p* = 0.53, two-sided); nor was time spent as a vaccination provider (*p* = 0.47, two-sided) or frequency of midwives vaccinating Aboriginal children (*p* = 0.53, two-sided). Although a high proportion of midwives (*n* = 41, 77.4%) reported feeling competent to raise Aboriginality status with their patients, 22.6% (*n* = 12) did not feel comfortable doing so.

### Questionnaire: attitude

Almost all midwives (*n* = 43, 94.6%) viewed vaccinations as a vital component of disease prevention. Approximately one-quarter of participants (*n* = 13, 23.2%) agreed with the statement that, “Delivering all scheduled vaccinations on the same day resulted in a greater number of side-effects, than if they were given on separate occasions” while 44.6% (*n* = 25) were uncertain. Approximately, 70% (*n* = 34) of midwives felt there was inadequate immunisation content on the needs of Aboriginal children within their undergraduate degree and 50% (*n* = 28) of participants were either uncertain or believed that some vaccines on the childhood immunisation schedule were ineffective. Although all midwives thought that it was important to discuss vaccinations with parents prior to discharge, 33.3% (*n* = 17) reported that they do not provide vaccine-related discharge education to new parents (Table 2). A total of 82.1% (*n* = 46) of midwives believed that having a greater selection of culturally appropriate promotional material available to distribute to parents and families at discharge would support their practice.

**Table 2:** Midwife response to attitude items in questionnaire

Statement	*n*	Response	Frequency	%
Some vaccines on the WA Childhood Immunisation Schedule are not very effective.	56	Agree	6	10.7
		Uncertain	22	39.3
		Disagree	28	50.0
Aboriginal children are at greater risk of contracting a vaccine-preventable disease than non-Aboriginal children.	56	Agree	45	80.3
		Uncertain	9	16.1
		Disagree	2	3.6
A delay in receiving scheduled childhood vaccinations is not a health risk as long as the child eventually gets them.	56	Agree	8	14.3
		Uncertain	9	16.1
		Disagree	39	69.6
Administering all scheduled vaccinations on the same day are more likely to result in a greater number of side-effects than if given on separate occasions.	56	Agree	13	23.2
		Uncertain	25	44.6
		Disagree	18	32.2
I feel that Aboriginal parents are hesitant about having their children vaccinated.	54	Agree	7	13
		Uncertain	22	40.7
		Disagree	25	46.3
I feel that Aboriginal parents are hesitant about specific vaccines	53	Agree	2	3.8
		Disagree	51	96.2
We are given clear direction from the WA Department of Health as to how we could improve the vaccination rates in Perth’s Aboriginal children	56	Agree	11	19.6
		Uncertain	17	30.4
		Disagree	28	50.0
By administering all scheduled vaccinations on-time some serious illnesses can be prevented.	57	Agree	56	98.2
		Uncertain	0	0
		Disagree	1	1.8
Vaccinations are a vital component of disease prevention in the community.	56	Agree	53	94.6
		Uncertain	2	3.6
		Disagree	1	1.8
Vaccinations may help to prevent children from developing some types of chronic disease.	56	Agree	52	92.9
		Uncertain	3	5.3
		Disagree	1	1.8
Vaccines are a safe way of protecting the health of Aboriginal children.	54	Agree	51	94.4
		Uncertain	3	5.6
		Disagree	0	0
Having a greater selection of culturally appropriate promotional material available to distribute to parents at discharge would support my role.	56	Agree	46	82.1
		Uncertain	7	12.5
		Disagree	3	5.4
Do you feel there was adequate content on the immunisation needs of Aboriginal children in the degree that enabled you to clinically practice?	48	Agree	14	29.2
		Disagree	34	70.8
It is important to discuss childhood Vaccinations with new parents prior to discharge.	55	Agree	55	100
		Uncertain	0	0
		Disagree	0	0
I discuss information on childhood vaccines with parents prior to discharge.	51	Agree	34	66.7
		Uncertain	6	11.8
		Disagree	11	21.5

### Questionnaire: subjective-norm

Approximately 62% of the midwives (*n* = 34, 61.8%) reported that they do not feel pressure from the general public to raise the vaccination coverage rates of Aboriginal children. When asked whether they felt that a *strong voice* from the Aboriginal community to improve vaccination services provided to Aboriginal children, 72.7% (*n* = 40) were either uncertain or did not agree. Almost one-half of all midwives (*n* = 24, 44.4%) did not feel that there was a high expectation from their management to vaccinate Aboriginal children or infants on-time and more than one-third (*n* = 19, 34.5%) did not feel that management viewed the achievement of high immunisation rates in Aboriginal children, as a priority.

Almost all midwives (*n* = 47 of 51, 92.1%) reported that they were not encouraged by their management to try new strategies to address the low coverage rates; 68.5% (*n* = 37) of the midwives indicated that they were not influenced by colleagues’ vaccination discussions with parents, to do the same (Table 3). In terms of influence on vaccination practice in the maternity setting, the most commonly selected option was the midwives professional regulatory body (32.9% of total responses received), their nurse manager (NM) (24.1%), colleagues (17.7%), while 7.6% of the total responses reported that the hospital ward doctor was a key source of influence on vaccination practice.

**Table 3:** Midwife response to subjective-norm items in questionnaire

Statement	*n*	Response Options	Frequency	%
There is pressure from the general public to increase the vaccination coverage rates in Aboriginal children	55	Agree	21	38.2
		Uncertain	21	38.2
		Disagree	13	23.6
There is pressure from the Aboriginal community to improve immunisation services for Aboriginal children in Perth.	55	Agree	15	27.3
		Uncertain	32	58.2
		Disagree	8	14.5
At my workplace, there is a strong expectation from management that all childhood vaccinations for Aboriginal children are given on-time.	54	Agree	30	55.6
		Uncertain	16	29.6
		Disagree	8	14.8
My manager views the achievement of high immunisation rates in Aboriginal children as a priority.	55	Agree	36	65.4
		Uncertain	13	23.6
		Disagree	6	10.9
In my workplace, we are encouraged to try new strategies to increase the proportion of fully vaccinated Aboriginal children in our geographical work area	51	Agree	4	7.9
		Uncertain	27	52.9
		Disagree	20	39.2
I feel that my profession as a whole views the achievement of high vaccination rates among Aboriginal children as a priority.	55	Agree	38	69.1
		Uncertain	8	14.5
		Disagree	9	16.4
I feel that my colleagues view the achievement of high vaccination rates among Aboriginal children as a priority.	55	Agree	30	54.6
		Uncertain	17	30.9
		Disagree	8	14.5
My colleagues’ practice of regularly discussing vaccinations with Aboriginal parents has influenced me to do the same.	54	Agree	17	31.5
		Uncertain	14	25.9
		Disagree	23	42.6
In terms of vaccinating Aboriginal children, which following groups, influence your current clinical practices?^a^	53	Manager	19	24.1
		Doctor (ward)	6	7.6
		Colleagues	14	17.7
		Other	14	17.7
		Professional body	26	32.9
We are encouraged to try new strategies to increase the coverage rates of Aboriginal children in Perth.	51	Agree	4	7.92
		Uncertain	27	52.9
		Disagree	20	39.2

^a^Indicates that more than one response could be recorded by midwives to this question

### Questionnaire: perceived behavioural control

Almost one half of all midwives (*n* = 24, 44.4%) reported that they did not feel confident with their knowledge of the vaccines administered to Aboriginal children, 55.6% (*n* = 30) were confident; 50.9% (*n* = 27) agreed with the statement that regular data updates to inform them of the coverage rates of Perth’s Aboriginal children would better support their clinical practice; 24% (*n* = 12) of midwives believed that the technical services within their workplace, including access to computers, would prevent them from providing a greater vaccination service (Table 4). A total of 80.4% (*n* = 45) of midwives reported that they use the same hospital protocols to maximise the proportion of fully vaccinated Aboriginal children as they did with non-Aboriginal children.

**Table 4:** Midwife response to the perceived behavioural control items in questionnaire

Statement	*n*	Response Options	Frequency	%
I feel confident with my knowledge of the childhood vaccinations given to Aboriginal children (birth–4 years).	54	Agree	30	55.6
		Uncertain	6	11.1
		Disagree	18	33.3
I find that I don’t have the time in my workday to follow up on siblings who are overdue for their vaccinations.	50	Agree	25	50.0
		Uncertain	17	34.0
		Disagree	8	16.0
It would help my clinical practice if I was given regular data updates on the rate of fully vaccinated Aboriginal children in the (geographical) area that I work in.	53	Agree	27	50.9
		Uncertain	0	0
		Disagree	26	49.1
Regarding the previous question, if you answered yes, how frequently would you like to receive the data if possible?	27	Yearly	3	11.1
		Six monthly	6	22.2
		Quarterly	11	40.8
		Monthly	4	14.8
		Fortnightly	3	11.1
I feel comfortable raising the subject of Aboriginality status with parents of newborn babies and siblings.	53	Agree	41	77.4
		Uncertain	4	7.5
		Disagree	8	15.1
At my workplace, the technical resources prevent me from providing a more efficient immunisation service.	50	Agree	12	24.0
		Uncertain	19	38.0
		Disagree	19	38.0
In my workplace we use the same protocols and strategies to maximise the proportion of fully vaccinated Aboriginal children as we do with non-Aboriginal children	56	Agree	45	80.4
		Uncertain	6	10.7
		Disagree	5	8.9

### Questionnaire: intention

Almost one-quarter of midwives (*n* = 11, 21.2%), stated they do not provide parents of newborn infants with the contact details of vaccination service providers prior to patient discharge; 67.3% (*n* = 35) were either uncertain if an Aboriginal Health Worker (AHW) visited new parents to discuss vaccinations pre-discharge or stated that they did not visit parents; 42.3% (*n* = 22) of midwives reported not using specific techniques or hospital protocols to ensure that Aboriginal women are comfortable during hospitalisation (Table 5).

**Table 5:** Midwife response to the intention items in questionnaire

Statement	*n*	Response Options	Frequency	%
I provide new parents with the contact details of vaccination providers in their area of residence prior to discharge.	52	Agree	41	78.8
		Uncertain	3	5.8
		Disagree	8	15.4
An Aboriginal Health Worker visits new parents to discuss vaccination prior to discharge.	52	Agree	17	32.7
		Uncertain	20	38.5
		Disagree	15	28.8
I offer vaccinations to siblings of Aboriginal babies that I see in my workplace.	49	Agree	5	10.2
		Uncertain	7	14.3
		Disagree	37	75.5
It is important to discuss childhood vaccinations with new parents prior to discharge.	55	Agree	55	100
		Uncertain	0	0
		Disagree	0	0
I use specific techniques or hospital protocols to ensure that Aboriginal women are comfortable during their hospital stay.	52	Agree	30	57.7
		Uncertain	0	0
		Disagree	22	42.3

### Questionnaire: areas to assist staff practice in vaccination

Almost two-thirds of midwives (*n* = 34, 65.4%) reported that more consistent cultural safety training would improve their vaccination clinical practice; 65.4% (*n* = 34) reported that a greater number of educational sessions focusing on the vaccines administered to Aboriginal children would positively impact and support their role within the hospital setting. Over 80% (*n* = 46) of participants advised that having a greater selection of culturally appropriate vaccination promotional material available would be beneficial to support clinical practice.

## Discussion

Aboriginal children living in Perth, WA experience some of the lowest vaccination coverage rates across the nation. Although general practice has been instrumental in delivering the majority of all childhood and adult vaccinations across Australia, the vaccine journey for the infant starts at birth, and typically in the hospital setting [1–3, 6]. For midwives who deliver ante-natal care to prospective mothers, a considerable number of opportunities exist during this period to create vaccination demand through awareness. However, for Aboriginal women, early contact with a maternity provider, linking in with an ante-natal service for continuity of care, and receipt of antenatal care, particularly in the first trimester is variable [15]. In Perth, approximately 60% of pregnant Aboriginal women are seen by medical teams or midwives in the first trimester. Although ambitious efforts are made to bolster engagement during this period [16], there are several reasons for this disconnection from services including high parity, pregnancy at younger ages than non-Aboriginal women and residing in low socio-economic areas impacting access [7]. McBain-Rigg and Veitch [10] offer alternate reasons and suggest that ante-natal engagement is impacted by transportation issues, access affordability and cultural and language barriers.

The national and international literature is consistent in terms of the perceived views held by midwives on the utility of vaccines [1, 6, 17]. Midwives are collectively described as being supportive of their application and use. This outcome was also identified in our study findings with almost all midwives reporting that vaccines were a vital component of disease prevention and reduced the risk of acquiring certain chronic diseases. Most of the midwives in our study had been vaccinating Aboriginal children for more than 10 years which represented a degree of stability and an expected familiarity with vaccine administration. However, 70.8% reported that their undergraduate degree was inadequate in terms of the vaccine requirements of Aboriginal children, and almost one-half lacked confidence in their current knowledge of vaccines. This finding is of relevance considering previous studies have shown that midwives with sound vaccination knowledge are more likely to promote vaccines and conduct difficult conversations with parents who express uncertainty or hesitancy with vaccines [3, 18]. Similarly, those midwives that have a positive attitude to vaccines are more likely to recommend them; a vaccination recommendation by a midwife has also been shown to enhance vaccine uptake [1, 9].

The role, influence, and potential impact of midwives on vaccination provision and promotion cannot be under-estimated. From a global perspective, an expanded role for midwives working in French hospitals has been described by Massot and Epaulard as being an effective strategy to raise vaccination [1]. In this location, changes to vaccine authorisation have resulted in vaccines being administered by midwives to the newborn, the women and more broadly, the family unit. Although this practice does not currently occur in Perth, a similar application could be used to increase the vaccination rates in Aboriginal children and families, considering the extended family and kinship is commonplace. This could also impact the timeliness of vaccinations in all Aboriginal children [10].

Cultural safety is an important feature of engagement and equity in healthcare [18]. For Aboriginal families, it also improves the accessibility and acceptability of the available services [10, 18, 19]. From the provider perspective, it offers insight into past trauma and colonisation [17]. Cultural safety also provides a greater understanding of kinship and relationships in Aboriginal families, the women’s responsibility to her family, and her positioning with the land, country, and environment. The majority of all midwives in this study were undertaking regular cultural safety training. This finding was in contrast to a Perth-based vaccination study which identified that general practice staff had low levels of cultural safety training being performed [8]. Of note however in the current study was the finding that almost one-quarter of the midwives who responded to our questionnaire felt uncomfortable identifying whether a patient was of Aboriginal heritage. These findings may help explain the reasons why midwives were supportive of wanting to perform cultural safety training more frequently.

A finding that needs further exploration was that midwives reported using the same hospital protocols and strategies in non-Aboriginal women as they did with Aboriginal women. Mc Bain-Rigg and Veitch [10] describe this attempt at equality as *difference blindness* and suggest that it occurs where healthcare providers treat all patients the same despite inequities of age, gender or ethnicity. The use of an equal rather than equitable approach, may disadvantage some Aboriginal mothers as it may overlook the specific needs of the women following birth, during the hospitalisation and in the lead up to discharge [10]. In the birthing wards of the two studied hospitals, targeted strategies to increase the vaccination coverage in Aboriginal children had not been considered. Midwives also reported that their manager did not perceive timely vaccinations or high vaccination coverage in Aboriginal children to be a priority. However, this may also have been due to a general lack of awareness of the low rates. When asked about influence on their vaccination practice, a high proportion of midwives indicated that they would refer to their regulatory body for advice. Nearly one-quarter of the responses indicated their CNM. These findings may offer insight to hospital management when attempting to circulate and reinforce public health messaging to midwives regarding vaccination and to strengthen the likelihood of midwives providing vaccine education and promotion [6].

Following the birth of a child in the study location, it is the responsibility of the parents to make follow up appointments with their general practitioner. This occurs at 6 weeks post-delivery and coincides with the next scheduled vaccine on the WA Childhood Immunisation Schedule. However, as Mc Aullay et al. [15] advise, despite considerable investments made to improve access to primary health in this location, more than 50% of WA infants are not being seen in the GP setting in the early stages of their lives. To add further to the complexity of post discharge follow up by a GP or healthcare provider, approximately 20% of midwives in this study advised that they don’t provide Aboriginal women with the details of providers that administer vaccinations or link them into existing services. For Aboriginal women who may not be local or are residing in a location which is unfamiliar to them post discharge, this may serve as a barrier to getting the child reviewed post discharge and vaccinated.

The role of the Aboriginal Health Worker (AHW) in Australian hospital settings provides a unique opportunity to bridge cultural barriers and enhance clinical outcomes [20].

The AHWs offer a broad social skillset with a diverse scope of practice. However, previous studies have also outlined a degree of role ambiguity, inadequate support and training for their work, and potential for organisational conflict to occur between cultural obligations and employment requirements [20]. When midwives were asked whether the AHWs visited Aboriginal women on the maternity wards to discuss vaccines, 38.5% were uncertain and an additional 28.8% advised that they did not. Although this area was not explored in great detail within the questionnaire, the use of this health professional could be used to strengthen the capacity of midwives in providing key vaccine messaging to Aboriginal families, provide vaccine education and link up families with culturally appropriate health providers who administer vaccines post discharge. This model of care would be reliant on providing support to AHWs to strengthen capacity, provide ongoing education and offer opportunity to expand their role across the maternity hospital setting [21].

## Conclusion

The findings of this study suggest that greater awareness of the vaccination coverage rates in Aboriginal children and knowledge of childhood vaccines by midwives in the maternity hospital settings in Perth could better support parents in their child’s vaccine journey. Although midwives reported being supportive of vaccine utility, a lack of knowledge concerning the vaccines administered to Aboriginal children, coupled with some uncertainty in the effectivity of vaccines used, should be addressed. Increasing midwife’s knowledge of vaccines should also be prioritised to enable more effective education to be delivered to Aboriginal families and create greater vaccination awareness following hospital discharge. These strategies should increase demand and provide support to general practice who carry the burden of vaccine administration to most of the Australian population. While it is acknowledged that midwives form an invaluable and critical part of the WA health service during the birth of a child, an enhanced role that provides Aboriginal parents with culturally appropriate education to increase vaccine literacy and create greater vaccination demand following hospital discharge, cannot be over-estimated.

## Data Availability

The datasets generated and analysed for this article are not publicly available due the possibility of further academic research being conducted in this area but content surrounding them are available from the corresponding author on reasonable request.

## Abbreviations

AD: Professor Amanda Devine
AHCWA: Aboriginal Health Council of Western Australia
AHPRA: Australian Health Practitioner Regulation Agency
AHO: Aboriginal Health Officer
AHW: Aboriginal Health Worker
AIR: Australian Immunisation Register
ECU: Edith Cowan University
GP: General Practice
LA: Dr. Lesley Andrew
NACCHO: National Aboriginal Community Controlled Health Organisation
NIP: National Immunisation Program
NMBA: Nursing and Midwifery Board of Australia
RC: Ms. Rebecca Carman
TPB: Theory of Planned Behavior
WAAHEC: Western Australian Aboriginal Health Ethics Committee
WA DoH: Western Australian Department of Health
